# Efficacy and safety of minimal invasive surgery treatment in hypertensive intracerebral hemorrhage: a systematic review and meta-analysis

**DOI:** 10.1186/s12883-018-1138-9

**Published:** 2018-09-03

**Authors:** Yiping Tang, Fengqiong Yin, Dengli Fu, Xinhai Gao, Zhengchao Lv, Xuetao Li

**Affiliations:** 1 Department of Neurosurgery, The Second Affiliated Hospital of Kunming Medical University, Kunming, 650101 Yunnan Province China; 2 Priority Ward, The Second Affiliated Hospital of Kunming Medical University, No. 374 Dianmian Avenue, Kunming, 650101 Yunnan Province China

**Keywords:** Minimal invasive surgery (MIS), Hypertensive intracerebral hemorrhage (HICH), Conservative method, Craniotomy, Meta-analysis

## Abstract

**Background:**

Recently, minimal invasive surgery (MIS) has been applied as a common therapeutic approach for treatment of hypertensive intracerebral hemorrhage (HICH). However, the efficacy and safety of MIS is still controversial compared with conservative medical treatment or conventional craniotomy. This meta-analysis aimed to systematically assess the safety and efficacy of MIS compared with conservative method and craniotomy in treating HICH patients.

**Methods:**

PubMed, Embase, Web of Science, and Cochrane Controlled Trials Register were used to identify relevant studies on MIS treatment of HICH up to November 2017. This study evaluated Glasgow Outcome Scale (GOS) score, Activities of Daily Living (ADL) score, pulmonary infection rate, mortality rate, and rebleeding rate for patients who underwent MIS, or conservative method, or craniotomy. Subgroup analyses were performed to compare randomization versus non-randomization and large hematoma versus small or mild hematoma. Begg’s test and Egger’s test were used to determine the potential presence of publication bias.

**Results:**

Sixteen studies consisting of 1912 patients were included in this study to compare the efficacy and safety of MIS to conservative method or craniotomy. MIS contributed to a significant improvement on the prognosis of the patients comparing with conservative group or craniotomy group. Patients undergoing MIS had a lower mortality rate when compared to those receiving conservative method. Also, MIS led to a notable reduction of rebleeding rate and an effective improvement of the patient’s quality of life by contrast with craniotomy. No obvious difference was found in terms of the pulmonary infection rate among the comparisons of three treatment methods. Randomization is not the potential source of heterogeneity, but hematoma volume may be a risk factor for post-operative mortality rate. No statistical evidence of publication bias among studies was found under most of comparison models.

**Conclusion:**

This meta-analysis suggests that minimal invasive surgery is an efficient and safe method for the treatment of hypertensive intracerebral hemorrhage, which is associated with a low mortality rate and rebleeding rate, as well as a significant improvement of the prognosis and the quality life of patients when compared with conservative medical treatment or craniotomy.

## Background

Hypertensive intracerebral hemorrhage (HICH), a common neurosurgery disease, seriously endangers lives of elderly patients and produces heavy economic burden for families and society [1]. HICH generally results from hypertension-induced intracranial arterial, venous, and capillary ruptures, of which, the mechanical stress of hematoma on brain tissue is the most common reason [2]. HICH has been reported to account for 50–70% of all spontaneous intracranial hemorrhage (ICH), its morbidity and mortality both occupy the top among all types of strokes, more than 30% survivors suffer from varying degrees of disability [3, 4]. Worse, the incidence of HICH continues to rise with aged tendency of population [5]. A study reported that the HICH patients with a hematoma volume >  50 ml are of a greater probability of mortality and disability [6]. Based on the risks and harmfulness of HICH, it is urgently necessary to seek out an effective therapeutic strategy for curing the patients with hypertensive cerebral hemorrhage (HCH).

Although the renowned deleterious influences of HICH, there have been no significant breakthrough in therapeutic schedules hitherto [7]. Currently, conservative medical treatment and surgical evacuation are the main options for HICH treatment [7]. Surgical treatment can be roughly divided into conventional craniotomy and minimally invasive surgery. Conventional conservative method has been used to treat of HICH for a long time, however, which has not made great progress in recent years, and was related with high fatality rate and mortality rate [8]. Craniotomy is the major surgical treatment for HICH, which can eliminate hematoma relatively thoroughly since it is applied, however, several disadvantages should also be noted, including large trauma, general anesthesia, obvious impairment on brain tissues, high blood loss, long operation time, severe edema reaction, various complications, poor prognosis and curative effect [9, 10].

Therefore, conservative treatment and craniotomy of hematoma could not achieve a desired therapeutic effect for HICH treatment.

With the development of imaging technique, minimal invasive surgery (MIS) has been widely applied in the treatment of HICH patients recently, which can reach to the designated position accurately and establish a work channel for clearing hematoma. MIS has been proved to be superior to conservative treatment or craniotomy in some respects [11]: 1) reducing the damage to cerebral tissues and surgical trauma; 2) relieving hematoma compression by targeting hematoma region directly; 3) treating patients with intracranial deep hematoma; 4) accelerating removal of hematoma;5) lowering the mortality and side-effects, as well as improving surgical prognosis. However, some studies [9, 12, 13] showed that MIS did not decrease the mortality rate or improve the long-term outcomes comparing with conservative treatment or craniotomy. Therefore, until now, it is unable to draw an exact conclusion about the impacts of MIS on the curative effect of HICH patients. Due to above controversial conclusions, we performed a comprehensive systematic review and meta-analysis in this study to evaluate the safety and efficacy of MIS for treating HCH.

## Methods

This systematic review and meta-analysis was performed to assess the safety and efficacy of minimally invasive surgery treatment for hypertensive intracerebral hemorrhage in accordance with PRISMA statement [14]. No ethical review was required in this study.

### Literature search

Four international databases including PubMed, Embase, Web of Science, and Cochrane Controlled Trials Register (CCTR) were searched from the earliest date to November 2017. The following search terms were used in different combination: ‘hypertensive’, ‘hypertension’, ‘cerebral hemorrhage’, ‘putamen hemorrhage’, ‘intracerebral hemorrhage’, ‘intracranial haemorrhage’, ‘cerebral bleeding’, ‘minimally’, ‘endoscopic surgery’, ‘keyhole’, ‘small bone window’, and ‘stereotactic drilling’. All terms were searched as subject headings and keywords. Meanwhile, Back Tracking Method was performed to ensure the integration of the included literatures.

### Inclusion and exclusion criteria

#### Inclusion criteria

Inclusion criteria in this meta-analysis were as follows: 1. Research subjects: computed tomography (CT)-confirmed diagnosis of HICH; 2. Intervention and comparison: MIS comparing with other treatment methods, including craniotomy or conservative medical treatment; 3. Primary outcome: mortality rate, rebleeding rate, lung infection rate, and the difference in the score of therapeutic efficiency.

#### Exclusion criteria

Exclusion criteria were as follows: 1. Publication language: not in Chinese or English; 2. Publication type: in the form of abstracts, statements, proceedings, comments, and other unpublished grey literatures, or reviews, pathology reports, project designs, cell experiments, and animal studies. 3. Data requirement: unable to provide required data or with less data in duplicated literatures.

### Data screening and quality evaluation

Two reviewers independently identified all studies according to inclusion and exclusion criteria, and assessed the quality of eligible articles. In the event of any disagreements, consensus was reached by discussion with a third reviewer. Two reviewers independently extracted the following data from each study: name of first author, publication year, country, range of eligible cases, study design (random), the type of patients, hematoma volume, number of cases, gender, age, the type of minimally invasive surgery, guideline, outcome index. All data in the charts are converted into numeric data. The third researcher was responsible for checking the extracted data.

### Statistical analysis

The primary outcomes across study were calculated by the dichotomous variables, and the data of each trial were showed as a relative risk (RR) ratio with a 95% confidence interval (CI). RR > 1 and *p* <  0.05 indicated that the long-term prognosis, side effects, and mortality in minimally invasive group were higher than those in other two groups. For the significant efficiency, we used Glasgow Outcome Scale (GOS) score, and Activities of Daily Living (ADL) score. Good outcome was defined as GOS score > 4, and ADL score > 3. For all outcomes, heterogeneity was quantified via Cochran’Q statistics and I-squared (I^2^) statistics [15]. A probability value of *p* <  0.05 or I^2^ < 50% was judged as statistical heterogeneity, then a random-effect model was performed to analyze the pooled data; on the other hand, a fixed-effect model was used. In case of a study with uncertain methodological quality, a sensitivity analysis was conducted by eliminating the peculiar study. If all the results were reversed, the pooled result would be considered as with low sensitivity and high stability. Subgroup analyses were performed to compare randomization versus non-randomization and large hematoma versus small or mild hematoma. Begg’s test and Egger’s test were used to assess the potential presence of publication bias, and *p* > 0.05 was considered a low publication bias. All statistical analyses were performed using Statistical Analysis System (Version 9.0; SAS Institute, Cary, NC) and RevMan5 software (Cochrane Information Management System).

## Results

### Literature research

Initial comprehensive literature search identified 260 potentially relevant articles from PubMed (*n* = 44), Web of science (*n* = 40), EmBase (*n* = 162), and CCTR (*n* = 14). 81 studies were excluded as duplicates, 179 studies were remained. According to the inclusion and exclusion criteria, 160 articles were removed due to the following reasons: systematic reviews (*n* = 82), unrelated studies (*n* = 57), other reasons causing HCH (*n* = 17), case reports (*n* = 3), and animal assay (*n* = 1). Next, we reviewed the full-text of the remaining 22 studies, and 6 studies were eliminated based on other reasons: not exactly HICH (*n* = 4) and without available data (*n* = 2). Finally, 16 studies [5, 9, 16–29] were included in this meta-analysis.

### Characteristics of the included studies

A total of 16 studies, consisting of 1912 patients, were included in the meta-analysis. Six of the studies were published between 2003 and 2010 [18, 19, 24–26, 28]. Most of the patients were Chinese except for 69 Japanese. Cranial computed tomography (CT) scan was used as the puncture positioning method in all the included studies. All patients were diagnosed with one type of hypertensive intracerebral hemorrhage diseases, and had been undergone a minimally invasive surgery. Eight of the included studies were randomized controlled trials [5, 16, 19, 20, 23, 25, 26, 29]. Most of the studies provided the detailed information of cases, including the proportion of male, age, the level of high voltage in addition to T. Nakano’s report [24]. 388 patients in 5 studies were treated with MIS vs. conservative method [17, 19, 20, 23, 25], whereas 1085 patients in 8 studies were treated with MIS vs. craniotomy [5, 9, 16, 18, 21, 22, 27, 29], and 439 patients in 3 studies were treated with MIS vs. craniotomy or conservative method [24, 26, 28]. The protocol of the studies was approved by the 4th Cerebrovascular Disease Conference (*n* = 5), Ethics Committee of General Hospital of Beijing Military Region (*n* = 1), and intracranial hematoma minimally invasive puncture removal techniques standardized treatment guidelines (*n* = 1), while 9 studies were not mentioned guideline. The outcomes reported in the articles were mainly based on GOS score (*n* = 9), ADL score (*n* = 5), and NIHSS (*n* = 4). The detailed data are summarized in Table 1.

**Table 1:** Characteristics of the included studies

First author (year)	Country	Duration	Random trail	Type of patients	Hematoma volume (ml)	Comparison of treatment methods Number (Male, Age (year), Hematoma volume (ml))			Information of minimally invasive			Outcome
						Conservative group	Craniotomy group	Minimally invasive group	Method	Puncture positioning method	Guideline	
Bo Huang (2003) [28]	China	1998–2001	No	HICH	> 30	38 (57.9%, 62.1 ± 5.8, 35–128)	38 (68.4%, 56.7 ± 5.3, 38–120)	36 (72.2%, 60.3 ± 5.1, 32–139)	Minimally invasive evacuation	Cranial CT scan	The 4th Cerebrovascular Disease Conference	NIHSS
Gang Yang (2016) [5]	China	2012–2014	Yes	HCH	> 30	–	78 (55.1%, 59.77 ± 5.06, 30–180)	78 (66.67%, 60.18 ± 5.51, 35–180)	Minimally invasive intracranial hematoma	Cranial CT scan	The 4th Cerebrovascular Disease Conference	BI
Guodong Wang (2017) [23]	China	2015–2016	Yes	Hypertensive spontaneous ICH (basal ganglia)	> 30	60 (66.67%, 55.2 ± 5.6, 31–87)	–	60 (61.67%, 60.2 ± 7.3, 33–85)	Minimally invasive intracranial hematoma	Cranial CT scan	The 4th Cerebrovascular Disease Conference	NIHSS
Guoqiang Wang (2014) [22]	China	2009–2012	No	Hypertensive spontaneous ICH	> 30	–	114 (71.9%, 55.3 ± 11.1, 30–128)	84 (73.8%, 59.4 ± 14.5, 30–144)	Minimally invasive puncture and drainage	Cranial CT scan	Ethics Committee of General Hospital of Beijing Military Region	GOS
Huili Kang (2016) [27]	China	2012–2014	No	HCH (basal ganglia)	20–40	–	30 (46.67%, 48 ± 12, 20–40)	30 (50%, 50 ± 10, 20–40)	Minimally invasive removal	Cranial CT scan	–	GOS
Jinbiao Luo (2008) [25]	China	2004–2008	Yes	Hypertensive mild hemorrhage (basal ganglia)	10–30	39 (58.97%, 54.3 ± 10.1, 10–30)	–	36 (58.33%, 56.3 ± 9.2, 10–30)	Minimally invasive directional soft tube placement	Cranial CT scan	–	GOS, ADL
Pingbo Wei (2010) [19]	China	2007–2010	Yes	HICH	20–40	39 (56.4%, 40–77, 39 ± 8)	–	31 (54.8%, 39–76, 31 ± 8) 36(52.7%, 41–80, 31 ± 9)	Minimally invasive surgery	Cranial CT scan	Intracranial hematoma minimally invasive puncture removal techniques standardized treatment guidelines	GOS
Shuwu Lin (2004) [26]	China	1995–2003	Yes	HICH	–	134 (52.2%, 60.9 ± 10.6, 33.5 ± 23.1)	10 (20%, 62.1 ± 11.2, 32.3 ± 22.5)	134 (48.5%, 60.1 ± 10.8, 35.0 ± 23.5)	Minimally invasive puncture and drainage	Cranial CT scan	–	ADL
T. Yamamoto (2006) [18]	Japan	2002–2006	No	HCH	–	–	10 (80%, 54–82, 15.9)	10 (80%, 53–86, 22.3)	Endoscopic surgery	Cranial CT scan	–	GOS
T. Nakano (2003) [24]	Japan	2000–2001	No	HICH	–	32	11	6	Endoscopic surgery	Cranial CT scan	–	GOS
Wenjun Wang (2017) [21]	China	2012–2016	No	HICH	> 50	–	34 (82.35%, 56.0 ± 12.37, 35.3 ± 18.28)	70 (82.35%, 61.10 ± 12.10, 68.8 ± 13.42)	Minimally invasive puncture and drainage	Cranial CT scan	–	GOS
Xinghua Xu (2017) [9]	China	2009–2014	No	Supratentorial HICH	55.2 ± 28.4/55.9 ± 27.6	–	69 (66.7%, 53.8 ± 13.5, 55.9 ± 27.6)	82 (7.07, 52.9 ± 12.3, 55.2 ± 28.4)	Endoscopic surgery	Cranial CT scan	–	MRS score, GOS
Xueyuan Wang (2011) [20]	China	2004–2009	Yes	Hypertensive basal ganglia hemorrhage	20–35	30 (53.33%, 45.73 ± 11.64, 20–35)	–	32 (56.25%, 46.75 ± 10.55, 20–35)	Minimally invasive puncture and drainage	Cranial CT scan	The 4th Cerebrovascular Disease Conference	ADL
YF Yan (2015) [17]	China	2010–2013	No	Hypertensive basal ganglia hemorrhage	15–30	12 (58.33%, 47.75 ± 9.16, 22.42 ± 4.70)	–	13 (76.92%, 55.31 ± 9.97, 25.18 ± 4.15)	Neuronavigation-assisted minimally invasive	Cranial CT scan	–	GOS, NIHSS
Yi Feng (2016) [29]	China	2006–2013	Yes	HCH	< 60	–	91 (63.73%, 69.10 ± 10.26)	93 (60.21%, 66.35 ± 12.23)	Endoscope-assisted keyhole technique	Cranial CT scan	–	ADL
Zaiyu Li (2012) [16]	China	2007–2010	Yes	HICH	> 30	–	110 (74.55%, 45–79, 30)	102 (71.57%, 37–75, 30)	Minimally invasive puncture suction drainage	Cranial CT scan	The 4th Cerebrovascular Disease Conference	ADL, NIHSS

*HICH* Hypertensive intracerebral hemorrhage, *HCH* hypertensive cerebral hemorrhage, *ICH* intracranial hemorrhage, *CT* computed tomography, *ADL* Activities of Daily Living, *GOS* Glasgow Outcome Scale

### Effects of interventions

#### Comparison of GOS score

**Fig. 1:**
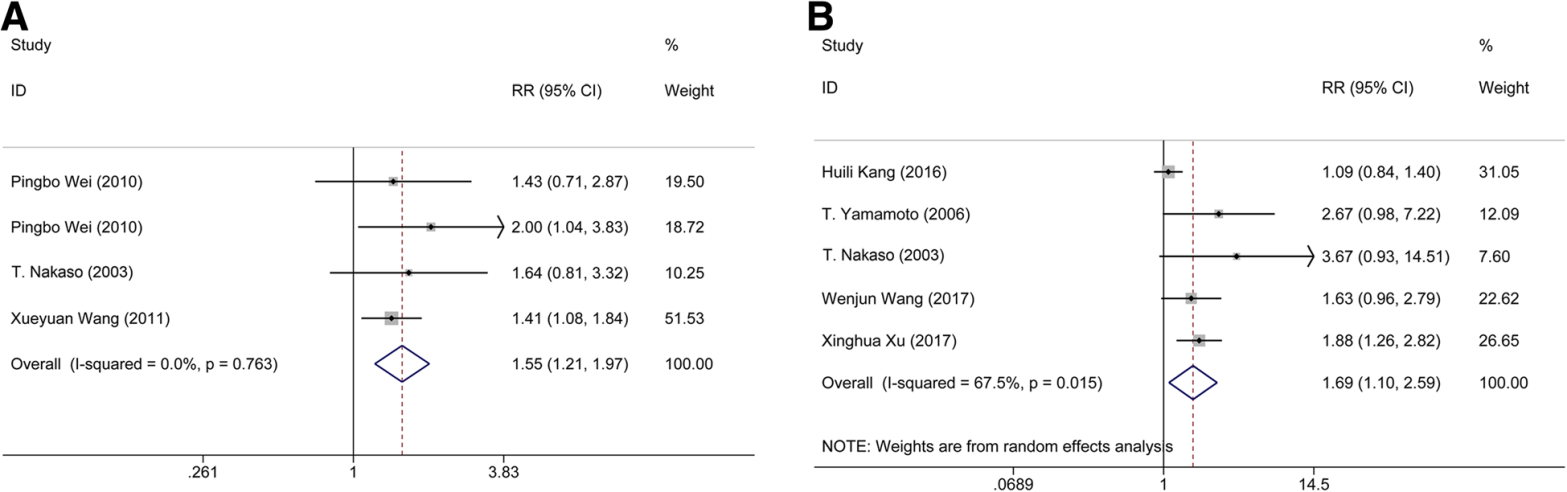
Comparison of GOS score. (**a**) Comparison of GOS score between minimal invasive surgery group and conservative group. (**b**) Comparison of GOS score between minimal invasive surgery group and craniotomy group

Data from 4 studies containing 258 patients were pooled to evaluate GOS score between MIS and conservative groups; meanwhile, 5 studies with data on 352 patients were available for the comparison between MIS and craniotomy groups. Heterogeneity (I^2^ = 62.1%, *p* = 0.032) existed in the GOS Score comparison between MIS and craniotomy groups, therefore, the random-effects model was used. The following results showed that MIS would lead to a statistical significance comparing with conservative group (*n* = 258; RR: 1.546; 95% CI: 1.121 ∼ 1.972; *p* <  0.001; Fig. 1a) or craniotomy group (*n* = 352; RR: 1.678; 95% CI: 1.099 ∼ 2.590; *p* = 0.017; Fig. 1b), suggesting that MIS shows a positive effect on the prognosis of the patients.

#### Comparison of pulmonary infection rate

**Fig. 2:**
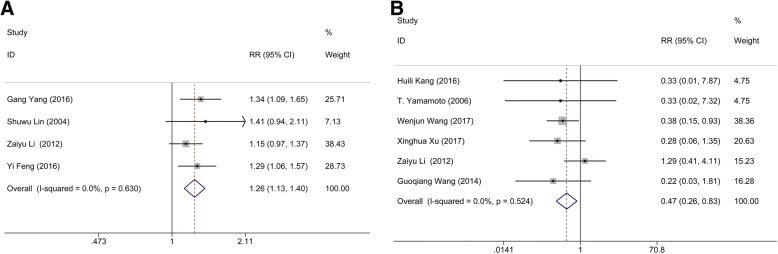
Comparison of pulmonary infection rate. (**a**) Comparison of pulmonary infection rate between minimal invasive surgery group and conservative group. (**b**) Comparison of pulmonary infection rate between minimal invasive surgery group and craniotomy group

Four studies containing data on 282 patients pooled pulmonary infection rate for MIS and conservative groups; meanwhile, 3 studies consisting of 486 subjects were available for the comparison between MIS and craniotomy groups. Heterogeneity (I^2^ = 77.8%, *p* = 0.011) was found in the pulmonary infection rate between MIS and craniotomy, assessed using a random-effect model. Clearly, no significant difference was found between the MIS and conservative group (*n* = 282; RR: 0.610; 95% CI: 0.342 ∼ 1.086; *p* = 0.038; Fig. 2a) nor craniotomy group (*n* = 486; RR: 0.700; 95% CI: 0.430 ∼ 1.141; *p* = 0.449; Fig. 2b), suggesting that MIS treatment has no positive influence on the pulmonary infection rate of patients.

#### Comparison of mortality rate

**Fig. 3:**
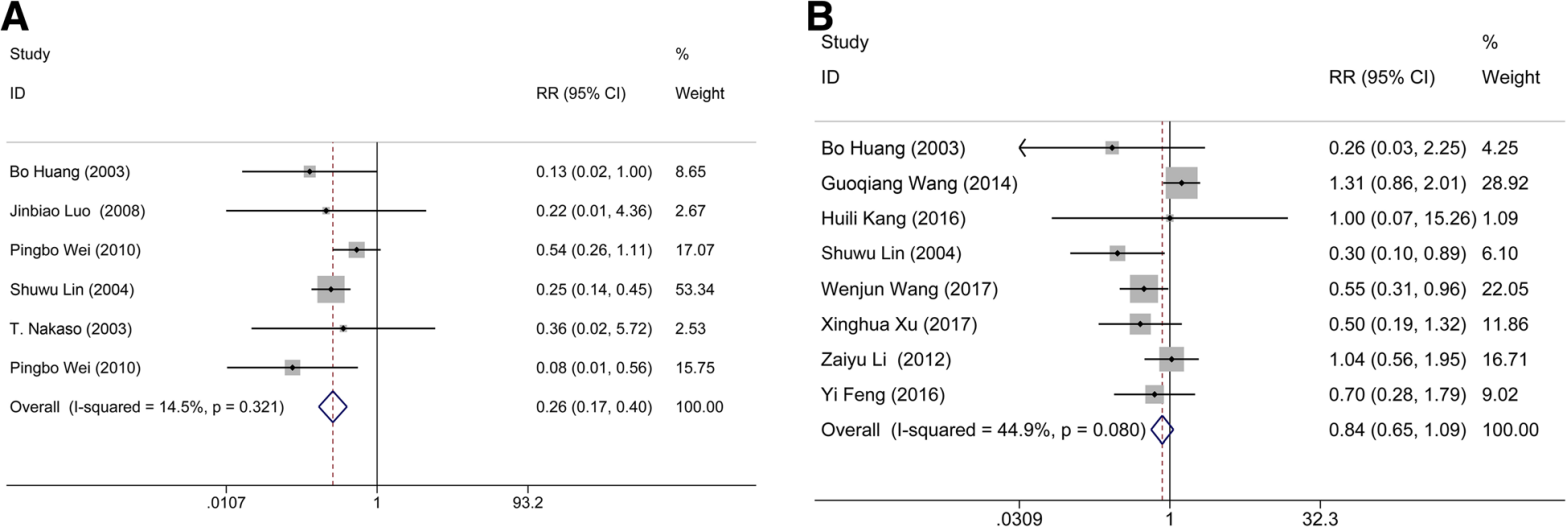
Comparison of mortality rate. (**a**) Comparison of mortality rate between minimal invasive surgery group and conservative group. (**b**) Comparison of mortality rate between minimal invasive surgery group and craniotomy group

Data from 6 studies with 600 patients were pooled to evaluate the mortality rate between MIS and conservative group; meanwhile, 8 studies consisting of 1127 subjects were available for the comparison between MIS and craniotomy groups. No heterogeneity occurred in either the comparison between MIS and conservative method (I^2^ = 14.5%, *p* = 0.321) nor craniotomy (I^2^ = 44.9%, *p* = 0.080). Obviously, apparent statistical significance was appeared in the pooled data between the MIS and conservative group (*n* = 600; RR: 0.265; 95% CI: 0.173 ∼ 0.404; *p* <  0.001; Fig. 3a), but not craniotomy group (*n* = 1127; RR: 0.839; 95% CI: 0.649 ∼ 1.086; *p* = 0.182; Fig. 3b), suggesting that MIS treatment could yield a lower mortality rate than conservative method.

#### Comparison of ADL score

**Fig. 4:**
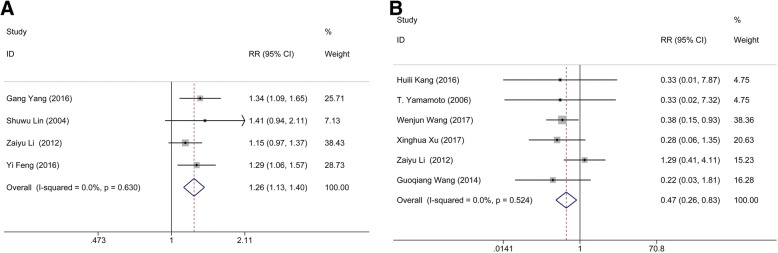
Comparison of ADL score and rebleeding rate. (**a**) Comparison of ADL score between minimal invasive surgery group and conservative group. (**b**) Comparison of rebleeding rate between minimal invasive surgery group and craniotomy group

Four studies consisting of 696 subjects were available for the comparison between MIS and craniotomy groups. There was no heterogeneity (I^2^ = 0.0%, *p* = 0.630) in the comparison of ADL score between MIS and conservative method. The following results showed that MIS had a statistical significance comparing with craniotomy group (*n* = 696; RR: 1.259; 95% CI: 1.133 ∼ 1.400; *p* <  0.001; Fig. 4a), indicating that MIS treatment can effectively improve the patient’s quality of life.

#### Comparison of rebleeding rate

Six studies containing 745 subjects pooled the data of rebleeding rate for MIS and craniotomy groups. No significant heterogeneity (I^2^ = 0.0%, *p* = 0.524) was found between these articles. The results showed that the rebleeding rate of the patients in MIS had a statistical significance comparing with that in craniotomy group (*n* = 745; RR: 0.468; 95% CI: 0.263 ∼ 0.832; *p* = 0.001; Fig. 4b), suggesting that MIS treatment can effectively reduce the postoperative rebleeding rate.

### Subgroup analysis

**Fig. 5:**
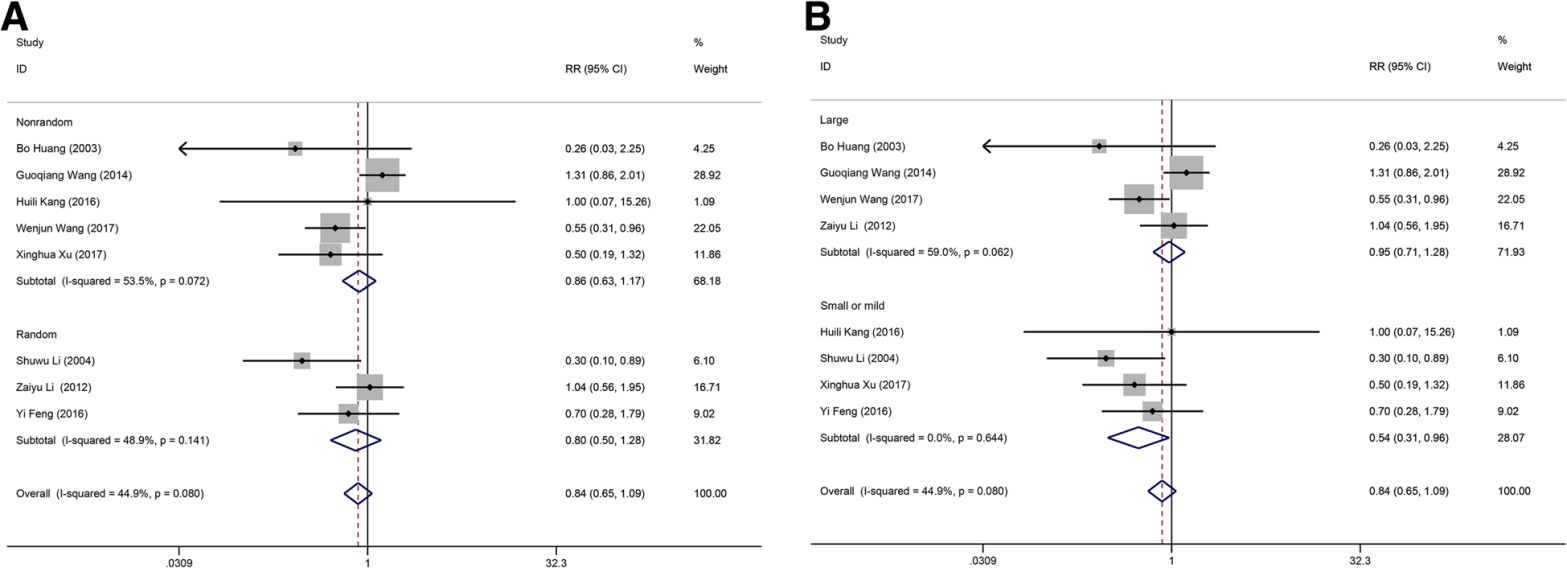
Subgroup analysis of the mortality rate between minimal invasive surgery group and conservative group in randomization and hematoma volume. (**a**) Subgroup of randomization. (**b**) Subgroup of hematoma volume

The data of heterogeneity analysis suggested that significant heterogeneity existed in the comparisons of GOS score between MIS and conservative method, and pulmonary infection rate between MIS and craniotomy. Based on the results of Table 2, we supposed that the sources of heterogeneity included the randomization of experiment design and the hematoma volume of HICH patients. Therefore, subgroup analyses were performed by stratified the status of randomization, and hematoma volume. The result of subgroup analysis based on the randomization of experiment design suggested that randomization would not change the pooled outcome: randomization (RR: 0.80; 95% CI: 0.50 ∼ 1.28; *p* = 0.358) and no-randomization (RR: 0.86; 95% CI: 0.63 ∼ 1.17; *p* = 0.322), which implied that randomization is not the potential source of heterogeneity (Fig. 5a). Also, the subgroup analysis of hematoma was conducted according to the volume of hematoma (large hematoma: volume >  30 ml; small or mild hematoma: volume < 30 ml). No significant difference of the mortality rate was found between MIS and craniotomy groups when the included cases with the hematoma volume >  30 ml (RR: 0.95; 95% CI: 0.71 ∼ 1.28; *p* = 0.755). Whereas, MIS would decrease the mortality rate of the HICH patients when the hematoma volume is less than a certain value (RR: 0.54; 95% CI: 0.31 ∼ 0.96; *p* = 0.035) (Fig. 5b). Above demonstrated that hematoma volume may be a risk factor for post-operative mortality rate. Nonetheless, more randomized controlled trial should be included to verify whether the above conclusion was correct or not because there was no clear record about the scope of hematoma volume in the included literatures.

**Table 2:** The pooled data

		RR (95% CI)	p of RR	I^2^	p of Heterogeneity	p of Begg’s test	p of Egger’s test
Minimally invasive group vs. conservative group	Rate of patients with a GOS score > 4 points	1.546 (1.121, 1.972)	< 0.001	0.0%	0.763	0.734	0.093
	Pulmonary infection rate	0.610 (0.342, 1.086)	0.038	0.0%	0.489	1.000	0.917
	Mortality rate	0.265 (0.173, 0.404)	< 0.001	14.5%	0.321	0.707	0.425
Minimally invasive group vs. craniotomy group	Rate of patients with a GOS score > 4 points	1.678 (1.099, 2.590)	0.017	67.5%	0.015	0.221	0.178
	Rate of patients with a ADL score > 3 points	1.259 (1.133, 1.400)	< 0.001	0.0%	0.630	0.308	0.336
	Pulmonary infection rate	0.700 (0.430, 1.141)	0.449	77.8%	0.011	0.296	0.08
	Rebleeding rate	0.468 (0.263, 0.832)	0.001	0.0%	0.524	1.000	0.656
	Mortality rate	0.839 (0.649, 1.086)	0.182	44.9%	0.080	0.386	0.132

*RR* relative risk, *GOS* Glasgow Outcome Scale, *ADL* activities of daily living

### Publication bias

Begg’s and Egger’s test were conducted to assess the publication bias of this meta-analysis, and the result was shown in Table 2. Obviously, there was no statistical evidence of publication bias among studies under most of comparisons, which suggested that our pooled data is of high authenticity and reliability.

## Discussion

Hypertensive intracerebral hemorrhage is one of the most common complications of hypertension. Currently, the minimally invasive surgery applied on the treatment of HICH has increased. The advantages of MIS include the well impermeability, less infection, low cost, low mortality and disability rates, better survival quality, and fast recovery time [11]. Although most reports about the curative effect of MIS are positive, some low level of recognition also existed, and the safety and efficacy of MIS remains unproven until now. Thus, it is great value to research application of minimally invasive surgery in hypertensive intracerebral hemorrhage. This systematic review with meta-analysis pooled the data from the included 16 studies concerning the effects of MIS, conservative method, and craniotomy on HICH to confirm the safety and efficacy of MIS. The main results suggested that MIS was associated with better prognosis outcomes and quality of daily living compared with conservative method or craniotomy. Moreover, this treatment modality could significantly decrease the mortality rate and rebleeding rate of patients. However, the incidence of pulmonary infection rate showed no significant difference between three groups. Overall, these findings demonstrated that MIS could be a safe and effective strategy in treating patients with HICH.

Rebleeding and pulmonary infection are two major complications related with the outcomes during treatments. Previous study [30] has suggested that MIS could decrease the risk of complications of patients comparing with traditional craniotomy for the following reasons: 1) MIS is associated with the smaller skin incision and shorter operation time; 2) craniotomy needs more space to operate, thus contribute to brain retraction, while MIS could reduce the risk of brain edema by affording preferable visualizing to the bleeding site due to without brain retraction. In our study, the patients underwent MIS exert low re-bleeding rate than those underwent craniotomy despite hemostasis cannot be easily performed under the direct vision during MIS. The reason for this possibly due to that hematoma is removed thoroughly in craniotomy approach, then result in a drastic pressure reduce in the hematoma cavity. The originally high pressure might cause the potential rebleeding in ruptured vessels. Although there is a gradual pressure reduction during the MIS treatment with continued hematoma drainage, it can in turn keep a steady pressure in the ruptured vessels and hematoma cavity, then promote the compression of hemostasis and stop the occurrence of rebleeding. An epidemiological study reported that pulmonary infection is one of the most common complications in HICH patients after treatment [31]. Conventional craniotomy is generally associated with significant blood loss, long-time anesthesia, and large trauma in elderly patients, which easily results in some complications, such as pulmonary infection. However, no significant difference was observed in the complication of pulmonary infection between the three groups, the result was in part agree with previously reported results [32]. Moreover, MIS was more effective in preventing death compared with conservative method but not craniotomy, which indicated that MIS was feasible in patients with HICH and its surgical efficacy was superior to that of conservative method can be achieved. In addition, we found a well-marked improvement of the prognosis and the life quality of the patients receiving MIS than those received craniotomy or conservative method, which verifies the long-term effect of MIS. The reason for these may be related with rapid and effective hematoma clearance of MIS. Puncture suction during MIS can remove hematoma rapidly, the hematoma-induced neurological damage could be relieved quickly, which lay a crucial foundation for improving the prognosis effect and living capability of patients in the future. Based on the above points, the advantages of MIS are prominent.

To further confirm whether the randomization status and the hematoma volume are the sources of heterogeneity, we performed the subgroup analysis. Subgroup analysis of randomization status found no difference in clinical outcomes in the treatment of HICH, suggesting that MIS treatment of hypertensive intracerebral hemorrhage is a safe and effective irrespective of the randomization status. Some researchers believed that the hematoma volume is an important factor for the patients received surgical treatments including MIS and craniotomy. For example, Zhou et al. [33] reported that MIS is suited to the hematomas with a volume of 25~ 40 ml, while, other forms of treatments like craniotomy should be performed when the volume hematomas > 40 ml. Meanwhile, the research of Yamashiro et al. [14] showed that MIS was associated with lower mortality rate when the mean hematoma volume of involved patients was at the range of 99 ~ 130 ml. In this subgroup analysis, no significant difference of the mortality rate was found between MIS and craniotomy groups when the included cases with the hematoma volume >  30 ml. Whereas, MIS of hematoma volume that is less than a certain value would contribute to a lower rate of death than other treatment options, demonstrating that hematoma volume may be a risk factor for post-operative mortality rate. However, due to a lack of sufficient evidence from the included literatures of the scope of hematoma volume, this underlying benefit of hematoma volume for HICH treatment requires more relevant studies to affirm. It is failed to perform to a subgroup analysis of the ethnic because most of the involved patients were Asians and the lack of related information from other races. Previous studies have confirmed that the incidence of HICH was varying in different races [3], which is mainly responsible for the differential gene expression [33]. As we known, minimally invasive surgery treatment is not belonged to the gene therapy. Thus, we believe that there is no significant difference in the therapeutic effect of MIS on HICH patients who have different ethnic backgrounds. Also, we did not conduct a subgroup analysis of the age. In this review, most of the included trials limited the age ≥ 30 and ≤ 80 years, thus the issue of MIS applying to the patients aged < 30 or > 80 years was ignored. Generally, the older patients are associated with a higher rate of mortality and the poorer prognoses [34]. However, no final verdict was achieved in terms of whether the older series undergoing MIS show worse outcomes than the young people. Zhou et al. [33] reported that the patients aged ≥30 years treated with MIS showed a significantly favourable outcome comparing with other treatment approaches, while no statistical difference was found in the patients aged ≥18 years. On the contrary, the study of Wang et al. [20] revealed that the older the patients received MIPD (minimally invasive puncture and drainage) is accompanied with the higher risk of death and the poor short- or long-term outcome. Here, we suspect that the older series may have better outcomes than the youngsters, reasons are as follows: elderly patients with an atrophic brain have a lower intracranial pressure when compared with the younger patients with same hematoma size, and they have more time to wait until the bleeding stop. Therefore, MIS will contribute to less brain retraction and brain tissue damage, with shorter anesthesia time and less blood loss in the elderly [29].

The main advantages of our study are as follows: First, this meta-analysis is based on the comprehensive literature search of several databases to confirm all associated comparative studies, and the research process was conducted by independent reviewers according to PRISMA statement. Second, most of identified literatures were published in more famous publications in recent years, which are of high-quality and contain more comprehensive content. Third, our study does not suffer from any publication bias, suggesting the high-reliability of our pooled data. Fourth, the large sample size provides some valuable data, which enables us to compare the outcomes by minimally invasive method, conservative method, and craniotomy, then summarizes some important conclusions. Fifth, this meta-analysis refers to all available clinically related outcomes, instead of selectively reporting only a few outcomes.

Also, several limitations in our meta-analysis should be taken into consideration: First, most of the involved studies were derived from the People’s Republic of China, which may restrict the applicability of our findings to some extent. Second, a few studies in our meta-analysis failed to provide the scope of hematoma volume, hence, we can’t be quite sure that the hematoma volume is a risk factor for post-operative mortality rate. Third, a lot of the included studies [1, 9, 17, 18, 21, 22, 24, 27, 28] on minimally invasive approaches to HICH were retrospective studies rather than RCTs. However, it also should be taken into account that it is very hard to carry out a prospective randomized study within a reasonable timeframe. Fourth, no studies provide the outcomes data of the side effects and the patient’s discharge from hospital, which are necessary to evaluate the safety of the MIS. Despite above, the findings in all studies generated unified results, as well as the similar surgical experience and postoperative outcomes, which reassures us that these disadvantages do not deny the validity of the meta-analysis.

## Conclusion

Collectively, based on the preliminary statistics and evaluation of the included 16 studies, it can be concluded that minimal invasive surgery is an efficient and safe alternative in the treatment of patients with hypertensive intracerebral hemorrhage, which has superior outcomes than conservative medical treatment or craniotomy. Although there is no improvement in pulmonary infection rate, MIS treatment is associated with the better prognosis and quality of daily living, as well as the lower mortality rate and rebleeding rate, when compared with conservative method or craniotomy. Hematoma volume may be a risk factor for post-operative mortality rate. However, more high-quality trials should be included before any claims can be putted forward.

## Abbreviations

ADL: Activities of Daily Living
CI: Confidence interval
CT: Computed tomography
GOS: Glasgow Outcome Scale
HCH: Hypertensive cerebral hemorrhage
HICH: Hypertensive intracerebral hemorrhage
ICH: Intracranial hemorrhage
MIS: Minimal invasive surgery
RR: Relative risk
